# Factors influencing future career interests of pharmacy interns in Saudi Arabia: a survey from 25 colleges of pharmacy

**DOI:** 10.1186/s12909-023-04022-9

**Published:** 2023-01-18

**Authors:** Abrar K. Thabit, Doaa I. Alghamdi, Reem O. Alaqi, Muath A. Alsufyani, Alaa A. Bagalagel

**Affiliations:** 1 Pharmacy Practice Department, Faculty of Pharmacy, King Abdulaziz University, 7027, Abdullah Al-Sulaiman Rd, Jeddah, 22254-2265 Saudi Arabia; 2 College of Pharmacy, Jazan University, Jazan, Saudi Arabia; 3 College of Pharmacy, Umm Al-Qura University, Makkah, Saudi Arabia

**Keywords:** Pharmacy, Pharmacists, Students, pharmacy, Career choice, Internship and residency, Saudi Arabia

## Abstract

**Background:**

Hundreds of pharmacists graduate from pharmacy colleges in Saudi Arabia, and various factors influence their choice of career pathway. Very few single-institution studies assessed career choices of pharmacy students with or without evaluating the influencing factors. Therefore, this study aimed to evaluate career choices and the associating factors of pharmacy interns from multiple colleges in Saudi Arabia.

**Methods:**

This was a cross-sectional study that surveyed pharmacy interns from 25 pharmacy colleges in Saudi Arabia using an online questionnaire. The survey was sent during the last rotation month in the internship year (May–June 2022).

**Results:**

Of 454 participants, 411 (90.5%) were enrolled in Doctor of Pharmacy programs. While most participants were interested in becoming clinical pharmacists (*n* = 183; 40.3%), a considerable number were also interested in working in different sectors of pharmaceutical companies and industry (*n* = 127; 28%). Internship training significantly correlated with selecting clinical pharmacy specialist career (*r* = 0.19; *P* = 0.0001), whereas salary/financial incentives significantly influenced the choice of working as sales and marketing representatives and pharmacy product specialists in pharmaceutical companies (*r* = 0.29 and 0.24; *P* < 0.0001 for both). College courses correlated with choosing academia in pharmaceutical sciences (*r* = 0.20; *P* < 0.0001), whereas summer training correlated with the community pharmacy career (*r* = 0.11; *P* = 0.02).

**Conclusion:**

Pharmacy colleges should utilize results from this study to enhance the exposure of pharmacy students during their academic years to different pharmacy career pathways by allowing the opportunity to shadow pharmacists from different sectors as part of college courses, inviting previous graduates, and activating the role of academic advisors in career orientation.

**Supplementary Information:**

The online version contains supplementary material available at 10.1186/s12909-023-04022-9.

## Introduction

Saudi Arabia is one of the largest and fastest-growing nations in the Gulf region. All divisions of the country have seen rapid growth, including health and healthcare services. Several healthcare sectors in Saudi Arabia hire Doctor of Pharmacy (PharmD) and Bachelor of Pharmacy or Bachelor’s degree in Pharmaceutical Sciences (BPharm) graduates, including hospitals, community pharmacies, universities, research centers, and pharmaceutical companies [1].

The number of pharmacy schools in Saudi Arabia has accelerated within the last decade reaching 23 public colleges and seven private colleges [2]. Two previous surveys from the year 2016 showed that the majority of pharmacy graduates were employed in the community pharmacy setting, accounting for 57% of the total workforce of 25,119 employees. The second-largest sector was factories, scientific offices, and drug stores, with a total of 22% of the workforce. Hospital pharmacies ranked third, with 19% of pharmacists were employed. Lastly, primary healthcare centers employed only 2% of pharmacists [1, 2].

In 2016, the Saudi Arabian government has set out a vision for transforming the country (Saudi Vision 2030). Vision's strategic goals include creating more jobs through increased participation of private and non-governmental organizations, raising the number of women in the workforce, and helping young graduates make better career choices [3].

Before the Saudi Vision 2030, a single-institution study was conducted among Saudi pharmacy interns. The study revealed that 34% (*n* = 41) of the participants preferred hospital pharmacy for their future career, while 25% (*n* = 30) chose academia, 25% (*n* = 30) were indecisive, 9% (*n* = 11) opted for owning a pharmacy, and another 9% (*n* = 11) preferred working in the pharmaceutical industry [4]. Moreover, another single-institution study done in 2017 showed that the preferred career pathway for Saudi female pharmacy students was hospital pharmacy in government hospitals (50.9%) followed by academia (19.4%), whereas community pharmacy was the least preferred pathway [5]. Another single-institution study found that work environment, advancement opportunities, and salary were the most important factors guiding career selection [6]. Despite the interesting findings of these studies, none investigated the reasons or influencing factors behind the career choices made by the participants.

As there is no large-scale, multi-institution study evaluating future pharmacy career choices and aspirations under the influence of the Saudi Vision 2030 and the factors influencing these choices, this study aimed to fill this gap and assess the career choices of pharmacy interns (advanced pharmacy practice experience [APPE] students) and the enablers that influence their decisions. Findings from this study can be used to develop recommendations for pharmacy education institutions and faculty to be involved in the process of career pathway decision making of prospective PharmD graduates.

## Methods

### Study design and participants

This was a cross-sectional study that surveyed pharmacy interns across Saudi Arabia using an online self-administered web-based questionnaire. The survey aimed to evaluate the choice of a post-graduation pharmacy career and the factors that influenced this choice. Participants were reached via direct contact with class leaders and school officials, where a link to the electronic survey was provided. The survey was sent during the last rotation month in the internship year (May to June 2022) to ensure that the interns had full exposure to different pharmacy sectors through their internship rotations, summer training, and curricular courses, as well as had a chance to speak to their academic advisors prior to graduating. Due to lack of contacts in 5 of the 30 colleges of pharmacy in Saudi Arabia, we were unable to distribute the survey to the interns of these colleges; however, we were able to distribute it to the interns’ class leaders of the remaining 25 colleges. The link was not shared on social media to avoid unsolicited participation from groups other than the targeted group of pharmacy interns in Saudi Arabia, such as non-intern pharmacy students. The study was approved by the Research Ethics Committee of the Faculty of Pharmacy, King Abdulaziz University, Jeddah, Saudi Arabia (Ref. PH-1443–49) and was performed in accordance with the relevant guidelines and regulations.

### Survey instrument

The online survey comprised 11 items (Supplementary material). It was developed by pharmacy interns and a faculty member, and then reviewed by another faculty member to establish content validity. Both faculty members are also clinical preceptors and academic advisors; hence, they have an experience in career advising for pharmacy students. As the survey included several multiple-choice questions rather than dichotomous and scaled questions, Cronbach’s alpha could not be calculated to evaluate the degree of reliability. Moreover, given the cross-sectional nature of the study, test re-test reliability could not be assessed. To avoid potential duplication of responses, the first question in the survey asked for students’ university identification numbers, which were kept confidential. The following four questions were basic about gender, type of pharmacy program, type of university, and the name of the university. The next five questions focused on career choice and were multiple-choice questions. The list of career choices was adapted from the American Pharmacists Association’s (APhA) list of career pathways [7, 8]. The last question was an open-ended question to add a qualitative explanation to the answers to previous questions. Answers that shared similar patterns were categorized accordingly for comparison purposes.

### Statistical analysis

Based on an estimated total number of pharmacy interns in Saudi Arabia of 1,200 interns, 428 responses were needed to meet a confidence level of 99% with a margin of error of 5%. Data were presented descriptively using numbers and percentages. Pearson correlation was used to evaluate the strength of the relationship between different demographic and influencing factors with career choices. Statistical significance was indicated with a *P* value of < 0.05. SPSS version 24.0 software (SPSS, Inc., Chicago, Illinois, USA) was used for analysis.

## Results

A total of 454 participants completed the survey with an estimated response rate of about 40% based on a total number of 1,151 interns of the included colleges who had their first attempt of the Saudi Pharmacist License Examination in year 2022 [9]. Table 1 lists the general characteristics of the participants. More than half of the participants were females (56.4%), and the majority studied in public educational institutions (88.1%). Furthermore, 411 (90.5%) of the participants were enrolled in PharmD programs while only 43 (9.5%) were enrolled in BPharm programs. Most of the responses came from central region universities (34.9%), whereas the least number of responses were from the northern region (5.1%). Participants had a median of two factors that influenced their career choices.

**Table 1:** General characteristics of the pharmacy interns and their educational institutions (*n* = 454)

**Characteristic**		**N (%)**
Gender	Female	256 (56.4)
	Male	198 (43.6)
Type of pharmacy program	PharmD	411 (90.5)
	BPharm	43 (9.5)
Type of educational institution	Public	400 (88.1)
	Private	54 (11.9)
Geographical location		
Central region	King Saud University	39 (8.6)
	Qassim University	43(9.5)
	Shaqra University	20 (4.4)
	Almaarefah University	18 (4)
	King Saud Medical Univrsity of Health Sciences	12 (2.6)
	Princess Nourah Bint Abdul Rahman University	10 (2.2)
	King Faisal University	10 (2.2)
	Hail University	4 (0.9)
	Buraydah Colleges	2 (0.4)
	Riyadh Alelm University	1 (0.1)
Western region	King Abdulaziz University	47 (10.4)
	Taif University	44 (9.75)
	Umm Al-Qura University	27 (5.9)
	Battarjee Medical College	19 (4.2)
	Tiabah University	12(2.6)
	Ibn Sina Medical College	5 (1.1)
Southern region	Jazan University	52 (11.6)
	King Khalid University	21 (4.6)
	Al Baha University	8 (1.8)
	Najran University	5 (1.1)
Northern region	University of Tabuk	14 (3.1)
	Al-Jouf University	5 (1.1)
	Northern Borders University	4 (0.9)
Eastern region	Imam Abdulrahman Bin Faisal University	19 (4.2)
	Prince Sattam Bin Abdulaziz University	13 (2.9)
Number of influencing factors ^a^		2 [1-3]

*BPharm *Bachelor’s in Pharmacy, *PharmD *Doctor of Pharmacy

^a^Median [interquartile range]

**Fig. 1:**
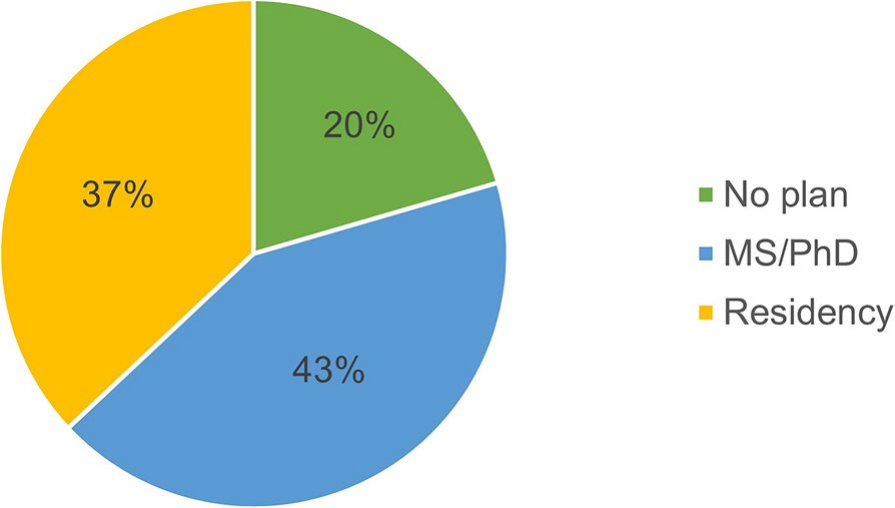
Intentions to pursue postgraduate education or training, MS, Master’s degree; PhD, Doctor of Philosophy degree

The prevalence of choice of different career pathways is shown in Table 2. While most participants were interested in becoming clinical pharmacists (*n* = 183; 40.3%) between general clinical pharmacy, specialized clinical pharmacy, ambulatory care, and academia, a considerable number were also interested in working in different sectors of pharmaceutical companies and industry (*n* = 127; 28%). Numerous factors influenced the participants to choose their careers (Table 3). The most prevalent factors were internship training, salary/financial incentives, as well as the influence of previous graduates. The distribution of the intention to pursue postgraduate education or training in pharmacy is represented in Fig. 1.

**Table 2:** Distribution of career pathway choices of pharmacy interns

Career Category	Career	N (%)
Clinical pharmacy	Clinical pharmacy specialist	109 (24)
	Ambulatory care clinical pharmacist	10 (2.2)
	General clinical pharmacist	9 (2)
Academia	Academia: Clinical practice	55 (12.1)
	Academia: Pharmaceutical sciences	18 (4)
	Academia: Economic, social, administrative sciences	5 (1.1)
Pharmaceutical company and industry	Pharmaceutical company: Sales & marketing	63 (13.9)
	Pharmaceutical company: Product specialist	41 (9)
	Corporate management	30 (6.6)
	Pharmaceutical industry: Research and development	16 (3.5)
	Pharmaceutical company: Supply chain	7 (1.5)
Health-system pharmacy	Health-system pharmacy: Inpatient	24 (5.3)
	Health-system pharmacy: Outpatient	17 (3.7)
	Health-system pharmacy: Drug information	5 (1.1)
	Health-system pharmacy: Compounding	3 (0.7)
Community pharmacy	Community pharmacist	18 (4)
	Community pharmacy management	6 (1.3)
Other	Research laboratory	2 (0.4)
	Pharmacovigilance, pharmacoepidemiology	2 (0.4)
	Military	2 (0.4)
	Pharmacy benefit management	1 (0.2)
	Will not work in pharmacy/Change career	9 (2)
	Indecisive	2 (0.2)

**Table 3:** Prevalence of different influencing factors

Influencing factor	N (%)
Internship training	315 (69.4)
Salary/financial incentives	165 (36.3)
Previous graduates'	138 (30.4)
Family and/or friends	96 (21.1)
Social media	81 (17.8)
College courses	80 (17.6)
Lecture conferences	73 (16.1)
Academic advisor/mentor	68 (15)
Summer training or part-time work	66 (14.5)
Personal choice	3 (0.7)
Comfortable environment	3 (0.7)
Matches personality	3 (0.7)
Future opportunities	1 (0.2)

Some participants selected more than one factor

Correlation analysis showed that internship training, learning about the career in a lecture or conference, and the impact of the academic advisor/mentor significantly correlated with selecting clinical pharmacy specialist career (*r* = 0.19, 0.15, and 0.11; *P* = 0.0001, 0.002, and 0.02, respectively); though, salary/financial incentives had a negative correlation (*r* = -0.20; *P* < 0.0001) indicating its low importance. In contrast, salary/financial incentives significantly influenced the choice of working in pharmaceutical companies as sale and marketing representatives (*r* = 0.29; *P* < 0.0001), as well as pharmacy product specialists (*r* = 0.24; *P* < 0.0001). Selecting these two pathways was also correlated with previous graduates’ experiences and opinions (*r* = 0.29 and *r* = 0.23; *P* < 0.0001 for both correlations). College courses was a major influencing factor for choosing academia in pharmaceutical sciences (*r* = 0.20; *P* < 0.0001), whereas summer training correlated with the community pharmacy career (*r* = 0.11; *P* = 0.02). The influence of family and friends and social media on all career choices was negligible, except for community pharmacy management, where social media had a slight correlation (*r* = 0.15; *P* = 0.002).

When asked about the intentions to change their career to other than a career in pharmacy, only 47 (10.4%) had such intentions vs. 89.6% who would work in the pharmacy field. Nevertheless, when asked whether pharmacy would be chosen again if a participant had the opportunity to start over, the majority (70.3%) said they would choose pharmacy again compared with only 29.7% who would not choose it again. The most common drive to re-choosing pharmacy was that pharmacy was the participant’s interest and passion, as well as the variety of opportunities the field of pharmacy has to offer. On the other hand, the main reason stated by those who opted to not re-choose pharmacy was the lack of interest. Only seven participants claimed that studying pharmacy involved too much memorization, and a similar number said they chose pharmacy due to social pressure.

## Discussion

The numbers of pharmacists in Saudi Arabia are expected to rise with the increase in the number of pharmacy colleges. Deciding on the preferred career pathway is one of the major decisions a prospective pharmacist should make prior to graduation. The present multi-institutional study showed that clinical pharmacy was the most common pathway chosen by pharmacy interns and that the choice was mostly influenced by internship training. A similar finding was reported in a study from Sudan, where the clinical pharmacy was ranked the top selected career path (30% of 220 survey respondents), which was influenced by previous training experience (80%) [10]. Interestingly, the study also found that curriculum content was another major influencing factor (70%) unlike our study that found that such factor had an impact on career choice of only 17.8% of the participants. A study from a large pharmacy college in Saudi Arabia found that work environment, advancement opportunities, and salary were the major factors influencing career choices [6]. Studies from Saudi Arabia and China demonstrated that salary, advancement opportunities, and a motivating environment for growth were particularly important to males, whereas a flexible and stable workplace was important to females [11, 12]. Previous studies from Saudi Arabia and abroad found that most pharmacy students opt to work as clinical pharmacists, hospital pharmacists, or community pharmacists, followed by preferences to work in other sectors, such as academia, research laboratories, factories, and pharmaceutical companies [13–15]. Such findings corroborate with observations from our study, except that community pharmacy was ranked very low with only 4% selecting it as a career choice. A study from Jordan, however, found that academia and research were the most preferred choices [16]. Structured internships in pharmacy colleges typically last 8 to 12 months, during which time students gain experiences in a variety of pharmacy fields, including clinical pharmacy, hospital pharmacy, community pharmacy, and pharmaceutical companies or factories [17]. Pharmacy interns get to have field training and shadowing during the internship year, unlike theoretical study during didactic years. Therefore, it was not unexpected to find it the top influencing factor in our study.

Introducing career pathway exploration to first year pharmacy students was evaluated in a study of 508 students who were given a four-week course to describe in-depth various pharmacy career pathways [18]. The authors reported a change from initial career plans in 50.8% of the students with many indicating that they would select different elective courses and seek new training opportunities compared with the students’ report prior to the course (*P* < 0.001). Early training in direct patient care, such as that offered in summer training or as part of the college curriculum, was shown to improve pharmacy students' knowledge, confidence, and skills in communicating with and caring for patients. Moreover, students found such training to be a positive and meaningful experience, which emphasized their desire to pursue a career in clinical practice [19–21]. The fact that only 14.7% of the study participants indicated that summer training guided their career choice suggests the need to put more emphasis on early pharmacy training to maximize its benefit and impact. Pharmacy colleges that do not currently mandate summer training after junior and senior years are recommended to encourage their students to make use of their free time during the summer to be trained in any pharmacy-related field.

Most pharmacy colleges in Saudi Arabia have switched their undergraduate programs from the BPharm program to the PharmD program, owing to its comprehensive clinical component, which aligns with the Kingdom’s vision to enhance the quality of healthcare while reducing mistakes that may harm patients and impact the economy [9]. Despite that, it remains important to educate prospective pharmacists on non-clinical roles of a pharmacist. A set of recommendations were presented by Park, et al. to help prospective pharmacy graduates select appropriate career pathways. They recommended recognizing the skills, technical and non-technical, possessed by pharmacy graduates prior to graduation, identifying unique career pathways to assist the students to explore potential alternatives, and educating the faculty and students prior to internship about job opportunities beyond the typical health-system setting [22].

Saudi Arabia is striving to expand its pharmacy services in various sectors, not only through increasing the number of undergraduates of pharmacy colleges, but also through increasing the number of professional clinical pharmacists and highly educated pharmacists. This has been accomplished by the fast growth of general and specialized pharmacy practice residency programs, as well as Master of Science (MSc) and Doctor of Philosophy (PhD) degree programs in different pharmacy fields [2, 17]. Currently, there are more than 22 hospitals that offer general pharmacy residency programs in Saudi Arabia, some of which also offer specialized programs in different medical specialties, such as critical care, oncology, and infectious diseases [17]. Nine pharmacy colleges in Saudi Arabia offer more than 25 MSc degree programs in various specialties, and one offers five PhD programs based on information listed on these colleges’ websites. The rapid development of such programs is crucial to graduate pharmacists with advanced knowledge and skills that could be utilized in different pharmacy-related jobs, which would ultimately improve the economy and provide better income to the pharmacists. Although the growth of pharmacy residency and graduate pharmacy programs has been noticeable, less attention has been given to pharmacy fellowship programs, which have a great emphasis on research. Research fellowship programs were originally developed in the United States, where many Saudi pharmacists obtained their postgraduate training, including completing fellowship training [23]. As only one pharmacy fellowship program is available in Saudi Arabia, it is suggested that well-established pharmacy colleges develop and promote research fellowship programs (clinical or laboratory-based) to further enhance the multidimensional research skills of pharmacists who would work in clinical, research, or industrial settings. Such a step can help nationalize the pharmaceutical industry; hence, contribute to the Kingdom's 2030 vision.

Academic advising and mentorship can have a great influence on selecting future career pathways according to a systematic review by Chan, et al., which evaluated the impact of academic advising on career opportunities exploration and the feedback from advisees [24]. In fact, academic advising and mentorship can help students set educational objectives that would enable them to attain their career aspirations [25]. Additionally, when one pharmacy school mandated academic advising, students reported positive feedback regarding the experience compared with students who did not receive advisement when advising was not mandatory [26]. All these reports signify the paramount role of academic advising in career pathway selection. Therefore, it is recommended that pharmacy colleges consider promoting academic advising to improve its impact on career exploration and selection since it was only reported by 15% of the participants in this study as an influencing factor. On another note, the marketing of careers in different pharmaceutical fields by former graduates through conferences or invitations to speak at pharmacy colleges had an impact on numerous participants in the current study as shown in Table 3. Moreover, previous graduates and employers are also encouraged to promote a better understanding and explanation of their careers on social media since only 17.8% of participants reported being influenced by information they found on such platforms even though one survey found that pharmacy students use all common social media websites for professional and educational purposes [27].

Broadening the vision of pharmacy students regarding the various career pathways using the aforementioned strategies (training, academic advising, meeting with former graduates, etc.) may help increase the retention rate since a small fraction (10.4%) of respondents to the current survey reported intentions to change career to other than a career in pharmacy. The reporting of interest in continuing to practice pharmacy by a large proportion of participants (89.6%) confirms results from a global study of 1,423 pharmacy students, of whom 1,110 (78%) opted to practice pharmacy after graduation [28].

As this is the first large multi-institutional study exploring the career pathway interests of pharmacy interns in Saudi Arabia and the factors influencing them, it has a few limitations. While we utilized the APhA’s career pathways list, some pathways were not included in the survey due to their lack of applicability in Saudi Arabia, such as mail service and home health care. Another limitation with regards to the influencing factors is the potential overlap between different factors. For example, the factor ‘previous graduates’, which was intended to mean live meetings and discussions, can also fall under social media if such graduates promoted their careers on social media platforms.

## Conclusion

This study evaluated pharmacy career choices by pharmacy interns who have finished all formal education and training and correlated such choices with various potential influencing factors. In this study, internship training correlated with clinical pharmacy career choice, financial incentives correlated with sales and marketing careers, college courses correlated with careers in academia in pharmaceutical sciences, and summer training correlated with the community pharmacy career. Results from this study can be utilized by pharmacy colleges, faculty members, conference organizers, current pharmacists, and social media influencers of pharmacists to enhance the exposure of pharmacy students during their academic years to different pharmacy career pathways. Pharmacy colleges may allow the opportunity to shadow pharmacists from different sectors as part of college courses, inviting previous graduates, and activating the role of academic advisors in career orientation. Such recommendations may enhance the knowledge of pharmacy interns about the breadth of career choices available for them and change their attitude toward them. Future interventional studies based on findings of this studies, such as assessing the impact of academic advising or frequent meetings with previous graduates, are suggested.

## Supplementary Information

**Additional file 1**.

## Data Availability

The datasets are available on Open Science Framework at: https://osf.io/s2nex/?view_only=9a810a99d3de4c68b26fb89013cad438.

## Abbreviations

APhA: American Pharmacists Association
APPE: Advanced pharmacy practice experience
BPharm: Bachelor’s degree in Pharmaceutical Sciences
MSc: Master of Science
PharmD: Doctor of Pharmacy
PhD: Doctor of Philosophy
