# Supplementary material for: Factors influencing future career interests of pharmacy interns in Saudi Arabia: a survey from 25 colleges of pharmacy
Source: BMC Med Educ. 2023 Jan 18;23:35. doi: 10.1186/s12909-023-04022-9 (PMC9847190; doi:10.1186/s12909-023-04022-9)
Supplement: Supplementary file 1 — Additional file 1. [file 12909_2023_4022_MOESM1_ESM.docx]

**Pharmacy Interns' Career Interests Survey Questions**

| 1. Student ID^*^ | |
| --- | --- |
| 1. Gender | - Male - Female |
| 1. What is your pharmacy degree program? | - Doctor of Pharmacy (PharmD) - Bachelor's degree in Pharmacy or Pharmaceutical Sciences (BPharm) |
| 1. Career choice | - Academia: Clinical Practice - Academia: Economic, Social, and Administrative Sciences (ESAS) - Academia: Pharmaceutical Sciences - General Clinical Pharmacist - Clinical Pharmacy Specialist - Ambulatory Care Clinical Pharmacist - Health System Pharmacy: Inpatient - Health System Pharmacy: Outpatient - Health System Pharmacy: Drug Information - Health System Pharmacy: Compounding Pharmacy - Community Pharmacy - Chain Community Pharmacy: Management - Corporate Management - Research Laboratory - Pharmaceutical Company: Product Specialist/Medical Liaison - Pharmaceutical Company: Sale and Marketing - Pharmaceutical Company: Supply Chain - Pharmaceutical Industry: Research and Development (R&D) - Pharmacy Benefit Management (PBM) - Will not work in pharmacy (change career) - Other^**^ |
| 1. Is your college public (government) or private? | - Public - Private |
| 1. University/College | - King Abdulaziz University - Umm Al-Qura University - Taiba University - King Saud University - Taif University - Tabuk University - Hail University - Imam Abdulrahman Bin Faisal University - King Faisal University - Prince Sattam bin Abdulaziz University - King Khalid University - Ibn Sina College - Riyadh Alelm College - Princess Noura bint Abdul Rahman University - Al-Qassim University - Buraydah Colleges - Batterjee Medical College - Jazan University - Najran University - Shaqra university - Aljouf University - Al-Maarefah University - Northern Borders University - Albaha University - King Saud University for Health Sciences |
| 1. What may have influenced your decision for your career choice? (check all that apply) | - College courses - Summer training - Internship training - Mentor/Academic advisor - Social media - Family and/or friends - Previous graduates - Lecture/Conference - Salary - Other^**^ |
| 1. Do you plan on pursuing postgraduate education or training? | - Master’s and/or PhD degree(s) - Residency - I don’t plan to |
| 1. Are you going to practice pharmacy or change career? | - Pharmacy - Change career |
| 1. If you had the opportunity to return to the first year, would you still choose pharmacy? | - Yes - No |
| 1. Why did you choose yes or no in the previous question?^**^ | |

^*^ To avoid duplicate entries.

^**^ Open-ended.
